# Clinicopathological significance of the *p16* hypermethylation in multiple myeloma, a systematic review and meta-analysis

**DOI:** 10.18632/oncotarget.18742

**Published:** 2017-06-27

**Authors:** Huiqing Yu, Liejun Yang, Yunfeng Fu, Meng Gao, Ling Tian

**Affiliations:** ^1^ Department of Medical Oncology, Chongqing Cancer Institute & Hospital & Cancer Center, Chongqing 400030, China; ^2^ The Third Xiangya Hospital of Central South University, Changsha 410013, China

**Keywords:** meta-analysis, p16, methylation, multiple myeloma, tumor suppressor gene

## Abstract

It is well known that the loss of function of the *p16INK4A* gene is mainly caused by the hypermethylation of the *p16* gene; however, whether or not the inactivation is associated with the clinical significance of multiple myeloma (MM) remains elusive. A meta-analysis was conducted to quantitatively determine the role of the *p16* hypermethylation in the clinical significance of MM. We demonstrated that MM patients show much higher hypermethylation rates on the *p16* gene in bone marrow compared to normal individuals, as well as monoclonal gammopathy of undetermined significance (MGUS). The difference of aberrant *p16* hypermethylation between MM patients in advanced stage and MM patients in early stage is not statistically significant. Interestingly, the survival rate of MM patients with the *p16* hypermethylation is much shorter compared to those without the *p16* hypermethylation. Our results demonstrate that hypermethylation status of the *p16* gene may play a role in the progression of MGUS to MM, as well as worse survival in MM. The *p16* hypermethylation, which induces the loss of function of the *p16* gene that plays a critical role in the early tumorigenesis of MM.

## INTRODUCTION

Multiple myeloma (MM) is a clonal hematological cancer formed by malignant plasma cells and overproduction of monoclonal immunoglobulin [1]. Over the past decade, a number of approved drugs such as lenalidomide, bortezomib and thalidomide have demonstrated significant clinical benefits in heavily pretreated MM patients [2–5]. Unfortunately, most of MM patients still relapse; the investigation of potential drug target and biomarker is still essential to enhance the survival rate in the patients with recurrent MM or those refractory to chemotherapy. Based on previous reports, several prognostic biomarkers including β2-microglo-bulin, serum albumin, hemoglobin and cytogenetic aberrations in MM have been used in clinical settings [6–9]. Epigenetic aberrations, such as hypermethylation of CpG islands located within the promoter region of the gene could lead to transcriptional repression/inactivation of the gene and participate in a crucial role in the development and progression of MM [10, 11]. The loss of function due to aberrantly methylated (hypermethylation) of CpG islands in tumor suppressor genes was observed in many cancers including MM, and the effect of which is equivalent to gene deletion and mutation in tumorigenesis. The *p16* gene is one of the most common cancer suppression genes, which is hypermethylated in many tumors, including MM [12, 13].

The *p16/INK4A/CDKN2* gene belongs to a family of cell cycle-related genes located on chromosome 9p21. It encodes a protein that competitively interacts with cyclin-dependent kinase 4 protein (Cdk4), which compromises the connection of cyclin D1 and Cdk4 to facilitate transition from the G1 phase to the next stage of the cell cycle [14]. The inactivation of the *p16* gene is caused by hypermethylation in MM, the published positive rates of the *p16* hypermethylation in MM are remarkably diverse [15–17]. The heterogeneous reported results do need for further investigation and evaluation of the correlation between the hypermethylation status of the *p16* gene and MM. Therefore, a meta-analysis was conducted to quantitatively examine whether or not the epigenetic changes are indicated by a higher level of methylation of the *p16* affecting clinical significance with MM.

## RESULTS

**Figure 1.**
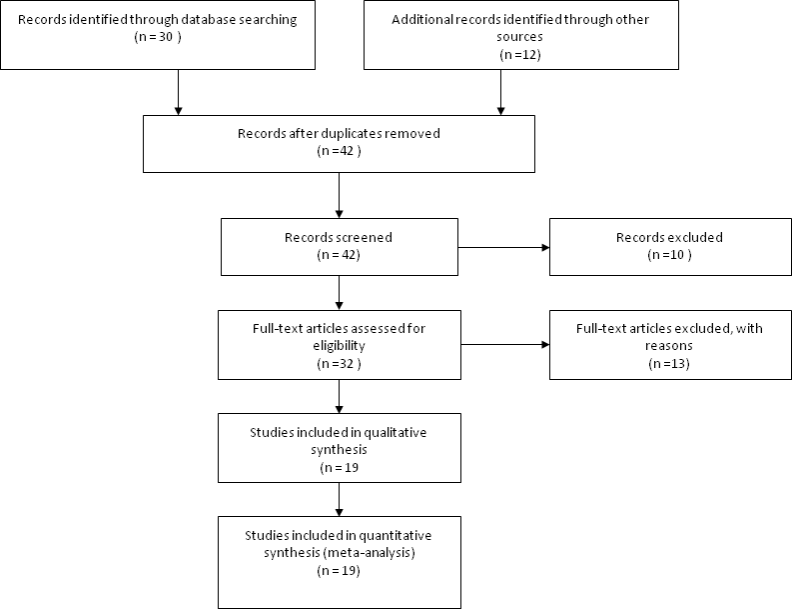
Flow chart of the study selection

Forty two articles were selected by the search method. The articles were excluded if they are reviews, *in vitro* or *in vivo* investigations, or studies unrelated to the topics. Finally, twenty four of articles were stripped out and this meta-analysis included nineteen studies (Figure 1).

### Study characteristics

Nineteen articles published from 1997 to 2013 and a number of 1348 patients from Argentina, South Korea, Brazil, China, Greece, Hong Kong, Germany, France, Spain, Austria, Poland, Japan and the United States were enrolled (Table 1).

**Table 1:** Basic characteristics of the included studies in multiple myeloma (MM)

Study	Country	Patients	Methods	Primary aim	Methylation site	Detectionof p16 protein
Kim et al 2013 [16]	South Korea	103	Methylation specific PCR (MSP)	Determine the methylation status of the *p16* gene and the clinical significance	Promoter, CpG islands	No
Park et al 2011 [17]	South Korea	99	MSP	Determine the methylation status of the *p16* and its association with common cytogenetic changes, clinicolaboratory findings	Promoter, CpG islands	No
Braggio et al 2010 [49]	Brazil	68	MSP	Determine the methylation status of nine tumor suppressor Genes including the *p16* in MM	Promoter, CpG islands	No
Stanganelli et al 2010 [41]	Argentina	44	MSP	Determine the methylation status of 7 genes including the *p16* in MM and MGUS	Promoter, CpG islands	No
Hatzimichael et al 2009 [50]	Greece	45	MSP	Aims to test CpG methylation of both *p16* and *TP73* occurs in MM	Promoter, CpG islands	No
Martin et al 2008 [51]	Spain	30	MSP	Determine the methylation status of 6 genes including the *p16* in MM and MGUS	Promoter, CpG islands	No
Chim et al 2007 [42]	Hong Kong	32	MSP	Determine aberrant *p16* promoter methylation in the progression of MM	Promoter, CpG islands	No
Gonzalez-Paz et al 2007 [18]	The United	522	MSP, RT-PCR	Investigate the biological and clinical implications of the *p16* gene methylation in MM	Promoter, CpG islands First exon,	Yes
Liang et al 2006 [52]	China	28	MSP, RT-PCR	Determine the methylation status of *p16* gene in MM	CpG islands	Yes
Seidl et al 2004 [25]	Austria	113	MSP	Determine the methylation frequencies of 10 genes including the *p16* in patients with monoclonal gammopathies.	Promoter, CpG islands	No
Galm et al 2004 [23]	The United States	56	MSP	Determine the methylation status of 11 well characterized tumor suppressor genes including the *p16* in MM.	Promoter, CpG islands	No
Chim et al 2004 [53]	Hong Kong	8	MSP	Determine the methylation status of 10 genes including the *p16* in MM	Promoter, CpG islands	No
Kramer et al 2002 [54]	Germany	48	MSP	Determine the frequency of Rb deletions, cyclin D1 alterations And hypermethylation of the *p16* in MM	Promoter, CpG islands	No
Chim et al 2003 [55]	Hong Kong	34	MSP	Determine whether or not disruption of the INK4/cyclin D-CDK/RB pathway is a common mechanism in the pathogenesis of MM	Promoter, CpG islands	No
Guillerm et al 2001 [56]	France	33	MSP	Determine *p15* and *p16* methylation in the progression of MM	Promoter, CpG islands	No
Ng et al 1997 [57]	Hong Kong	12	MSP	To investigate whether *p15* and *p16* deactivated by deletions, mutations, and hypermethylation in MM	Promoter, CpG islands	No
Tasaka et al 1998 [58]	Japan	16	MSP/RT-PCR	Determine the *p16* methylation in the progression of MM	Promoter, CpG islands	Yes
Guillerm et al 2003 [24]	Poland	61	MSP	Determine the *p15, p16* methylation in the outcome of MM	Promoter, CpG islands	No
Fu et al 2002 [19]	China	42	MSP	Determine the methylation status of the *p16* in MM and MGUS	Promoter, CpG islands	No

Note: MSP: Methylation specific PCR; Yes: p16 protein expression was detected; No: p16 protein expression was detected.

### The p16 hypermethylation and clinicopathological characteristics

#### The loss of the *p16* expression through hypermethylation in MM and *MGUS*

**Figure 2:**
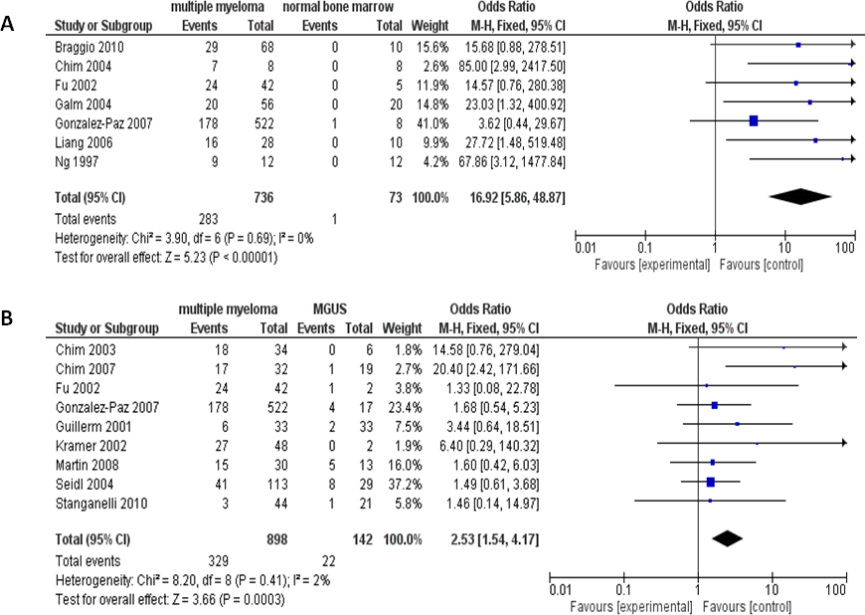
The pooled OR from 7 studies including 736 multiple myeloma (MM) and 73 normal bone marrow (OR=16.92, 95% CI=5.86-48.87, *p*<0.00001) **(A)**.The pooled OR from 9 studies including 898 MM and 142 monoclonal gammopathy of undetermined significance (MGUS) (OR=2.53, 95% CI=1.54-4.17, *p*=0.0003) **(B)**.

MM patients showed higher proportion of the *p16* hypermethylation compared to normal individuals. The pooled OR from 7 articles which include 736 MM and 73 normal bone marrow are presented in Figure 2A (odds ratios, OR=16.92, 95% confidence intervals, CI=5.86-48.87, *p*<0.00001), which demonstrates that the loss of *p16* expression by hypermethylation plays critical role in the tumorigenesis of MM. In addition, the *p16* hypermethylation also is detected in monoclonal gammopathy of undetermined significance (MGUS) and is remarkably less than in MM (OR=2.53, 95% CI=1.54-4.17, *p*=0.0003), as shown in Figure 2B. Due to limited studies of the *p16* methylation on both normal individuals and MGUS patients [18, 19], we are unable to compare the difference of the *p16* hypermethylation within these two groups of individuals.

### The *p16* hypermethylation in the progression of MM

**Figure 3:**
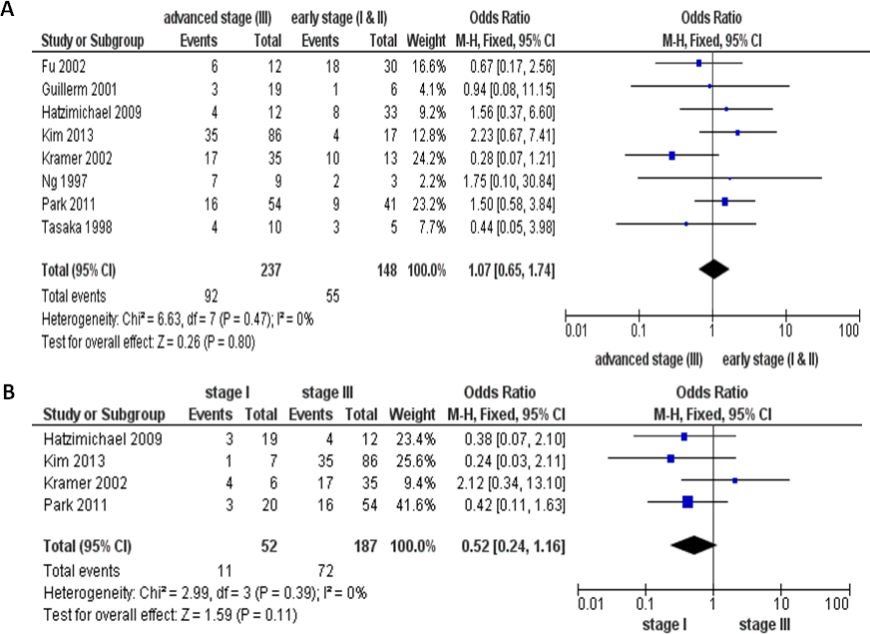
The pooled OR from 8 studies including 385 multiple myeloma (MM) patients Aberrant *p16* hypermethylation was not significantly higher in advanced MM (III) than that from early staged MM (I &II), OR=1.07, 95% CI=0.65-1.74, *p*=0.80 **(A)**. The pooled OR from 4 studies including 239 MM patients. The *p16* hypermethylation was also not significantly higher in stage III, than that from stage I, OR=0.52, CI=0.24-1.16, *p*=0.11 **(B)**.

We then analyzed 382 MM patients pooled in 8 investigations to evaluate if the role of inactivation of the *p16 via* hypermethylation on the progression of MM. In Figure 3A, aberrant *p16* hypermethylation is not remarkably higher in advanced MM (III) than that in early staged MM (I &II), OR=1.07, 95% CI=0.65-1.74, *p*=0.80. We further analyzed 239 MM patients pooled in 4 studies and found there is no significant difference between the level of the *p16* hypermethylation in stage III and stage I, OR=0.52, CI=0.24-1.16, *p*=0.11, as presented in Figure 3B. These results indicate that the inactivation of the *p16* gene due to hypermethylation may not play a critical role in MM development from initial stage to advanced stage.

### Prognostic significance of the *p16* hypermethylation in MM

**Figure 4:**
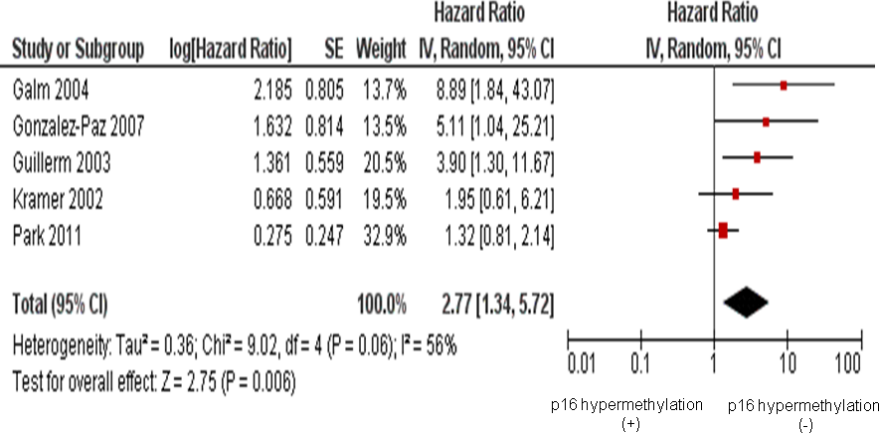
Five studies included investigated the relationship between overall survival (OS) and the *p16* hypermethylation The pooled HR for OS showed that the *p16* hypermethylation was associated with worse survival in multiple myeloma (MM) (HR=2.77, 95 % CI=1.34-5.72, *P*=0.006).

Five studies included investigated relationship between the *p16* hypermethylation and overall survival (OS). The pooled hazard ratios (HR=2.77, 95 % CI=1.34-5.72, *P*=0.006) for OS indicates that the *p16* hypermethylation is correlated with worse survival in MM as presented in Figure 4.

### Publication bias and sensitivity analyses

**Figure 5:**
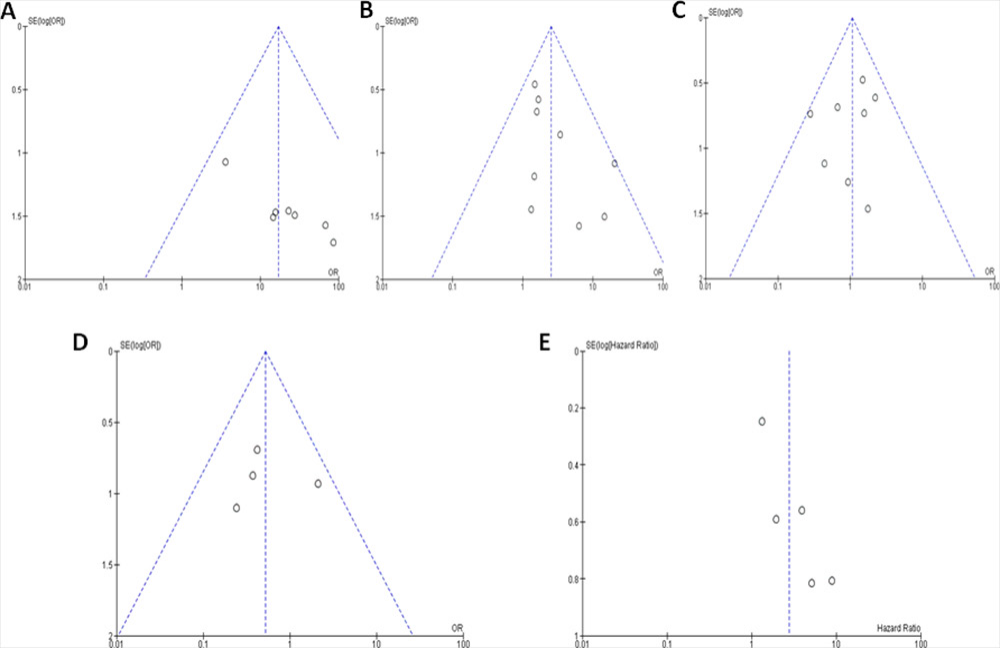
The funnel plots were largely symmetric suggesting there were no publication biases in the meta-analysis of the *p16* hypermethylation and clinicopathological features The funnel plot from 7 studies comparing multiple myeloma (MM) and normal bone marrow **(A)**. The funnel plot from 9 studies comparing MM and monoclonal gammopathy of undetermined significance (MGUS) **(B)**. The funnel plot from 8 studies comparing different staged MM patients (III VS. I &II) **(C)**. The funnel plot from 4 studies comparing different staged MM ( III VS. I) **(D)**. The funnel plot from 6 studies in determining overall survival (OS) and the *p16* hypermethylation in MM patient **(E)**.

In the current study of the *p16* hypermethylation and clinicopathological features using meta-analysis, the publication biases were evaluated by the funnel plots. The publication biases were ruled out by the symmetric funnel plot (Figure 5). The sensitivity analyses were conducted by removing one study at a time to determine the stability. The analysis results showed that the pooled ORs and HRs are not remarkably changed, suggesting the stability of these meta-analyses.

## DISCUSSION

Aberrant DNA methylation, which has been well characterized in many tumors, is believed to cause tumor formation, progression and worse prognosis [20–22]. The *p16* gene is the most common methylated gene in MM [23–26]. The loss of the *p16* function is significantly associated with the gene hypermethylated in a variety of cancers including MM [27–34]. Although there have been a number of studies are involved with the methylation level of the *p16* in MM, the pathological significances of inactivation of the *p16* in MM and clinical role are still elusive. The pooled data from this meta-analysis indicate that 1) remarkably higher the *p16* hypermethylation was detected in MM than that from normal bone marrow; 2) the *p16* hypermethylation was also detected in MGUS, but remarkably less than that from MM; 3) MM patients in advanced MM do not show high levels of the *p16* hypermethylation compared with those at early stage; 4) MM patients with the *p16* hypermethylation had a lower survival rate than those without the *p16* hypermethylation. The analysis evidences revealed that the *p16* hypermethylation proportion in MM was remarkably higher than that in the normal bone marrow, indicating that the *p16* hypermethylation may play a role in the initiation of MM. Based on the observation that the *p16* hypermethylation status are reversible by the demethylation agents, currently several agents have been applied in clinics to slow down the process of carcinogenesis and progression, therefore improve prognosis. Histone deacetylase inhibitor, sodium phenylbutyrate, and 5-Aza-2'-deoxycytidine (5-Aza-CdR) were reported to induce *p16* gene demethylation and tumor cell apoptosis in MM [35]. In another report, arsenic trioxide was also reported to induce *p16* gene demethylation and tumor cell growth inhibition in MM [36]. This strategy may bring hope for tumor treatment through gene manipulation and gene-targeted therapy. Due to limited studies of the *p16* methylation on both normal individuals and MGUS patients [18, 19], we are unable to compare the difference of the *p16* hypermethylation within these two groups of individuals. Nevertheless, we can still conclude that the *p16* hypermethylation status is associated with disease development and progression from benign MGUS to malignant MM and a stratification factor for patients with MM.

Recent studies show that MM is consistently preceded by a precursor state, MGUS [37–39]. Progression from MGUS to MM seems a result of the accumulation of genetic and epigenetic abnormalities, indicating a stepwise progression of alterations at genetic and epigenetic levels [40]. In fact, hypermethylation of a number of genes, such as *p15INK4B*, *ARF, p27 KIP1, SOCS-1, RASSF1A, death-associated protein* (DAP) kinase, non-receptor type 6 (SHP1), and *TP73* genes were reported in MGUS [41, 42]. Among six studies, only one study detected the *p16* gene hypermethylation in normal cohort (Figure 2A). In contrast, seven out of nine studies detected the *p16* gene hypermethylation in MGUS (Figure 2B). Since only one study has relevant data, we were not able to determine whether or not the *p16* gene hypermethylation in MGUS is significantly higher than that in normal cohort by meta analysis. Our results showed that significantly higher the *p16* hypermethylation was detected in MGUS and significantly less than that from MM. Hypermethylation of the *p16* gene could potentially be participants in the development of MGUS to MM.

The *p16* hypermethylation in MM was not observed to associated with advanced stage. We also did not find that the *p16* hypermethylation was remarkably higher in stage III, compared to that from stage I of MM. These evidences suggest that the *p16* hypermethylation could be an early event. Only five studies examined the correlation between the overall survival and the *p16* hypermethylation in MM, they showed homogenous results. The pooled HR (HR=2.77, 95 % CI=1.34-5.72, *P*=0.006) for OS showed that MM patients with high level of the *p16* hypermethylation have significantly shorter survival (Figure 4). We observed that the pattern of the *p16* methylation in different stages of MM were similar, however, several years after disease progression, MM patients with the *p16*-positive expression had remarkably improved survival rates compared to the *p16*-negative patients. There is no good explanation about that there is no significant difference of *p16* hypermethylation in initial and advanced stage MM, however, there is significant difference in overall survival in MM. Maybe *p16* hypermethylation status is more associated with stage- unrelated molecular changes and therapeutic response in MM patients. Galm et al [23] reported that hypermethylation of *p16* was correlated with a poor prognostic impact in MM patients. Our analysis further supports that detection of *p16* hypermethylation may provide an excellent prognostic marker for MM patients.

This study may have several potential limitations. The selection biases and unidentified confounders could not be completely excluded, since all of the included studies were experimental. Second, the identification of articles was based their publication only in English and Chinese, other articles with potentially meaningful data in other languages were not selected. Therefore, caution could be taken when our analysis are applied to the general populations.

In summary, this study using meta-analysis demonstrates that the *p16* hypermethylation may play a role in the progression of MGUS to MM, as well as worse overall survival in MM. The *p16* hypermethylation, which induces the loss of function of the *p16* gene, plays an important pathological role in the early carcinogenesis of MM.

## MATERIALS AND METHODS

### Search method and selection criteria

We searched Embase, Pubmed and ISI web of knowledge to select studies from June 1, 1996 to February, 2017 using the search terms: “multiple myeloma”, “plasma cell myeloma”, and “Kahler's disease”, “methylation”, and “*p16*”. We also checked the reference lists of the reviews and selected articles for related articles. Although our article or publication search did not have language limits initially, we only took into account studies published in English and Chinese for full-text reading and final evaluations. After the exclusion of the redundant and non-relevant publications from the different databases, the remaining papers were evaluated in the full text version for in- and exclusion criteria. Authors’ references of included studies were also checked for other related investigations. All searched data were retrieved. The most complete investigation was included if the same patient populations were published in different resources.

Criteria for identification that an eligible study has to meet were as follows: (1) the *p16* methylation and/or expression evaluated in primary MM, (2) the *p16* methylation determined by polymerase chain reaction (PCR), (3) researches revealed the relationship between the *p16* methylation and/or expression of MM clinicopathological parameters and prognosis, (4) studies which provided sufficient data to calculate Odds ratio (OR) and/or hazard ratio (HR) about overall survival (OS) and 95 % confidence interval (CI). The exclusion criteria: (1) reviews, letters, case studies, editorials, conference abstracts, expert opinion, (2) articles that had no information of OS or that could not calculate the HR about OS from the given information; and (3) all publications regarding *in vitro*/*ex vivo* studies, cell lines and human xenografts were also excluded. In addition, “aberrant” *p16* methylation or *p16* hypermethylation is defined by clear PCR product band detected by methylation specific PCR (MSP).

### Data extraction and methodological assessment

Two authors (HY, LY) independently reviewed and analyzed data from eligible studies. Two authors (YF, MG) evaluated all of publications according to the inclusion and exclusion criteria. The following criteria were chosen for each study: the first author name, year of publication, number of cases, sample source, methylation detection method, clinicopathological parameters, methylation rate, and/or expression, and follow up. Data for study characteristics were summarized in a table format. Investigation heterogeneity was evaluated to determine whether or not the data of the various studies could be analyzed for a meta-analysis.

Three investigators (LY, YF and LT) read through each publication independently for the methodological evaluation of the studies, and assessed and scored them according to REMARK guidelines and ELCWP quality scale [43, 44]. Then they provided the quality scores, compared them, and then reached a consensus value for each item.

### Statistical analysis

Analysis was performed using the STATA 12.0 (Stata Corporation, TX, USA) and Review Manager 5.2 (Cochrane Collaboration, Oxford, UK). The pooled frequency of the *p16* hypermethylation and 95% confidence intervals (95% CI) were estimated. The frequency of the *p16* hypermethylation was compared in different tumor characteristics. Heterogeneity among studies was examined with Cochran’s Q test [45] and the *I*^*2*^ statistic [46, 47]. A fixed effect model was used to calculate parameters, when heterogeneity was not an issue (*I*^*2*^ values <50%), while a random-effects model was used to pool data and attempt to identify potential sources of heterogeneity based on subgroup analyses, if there was substantial heterogeneity (*I*^*2*^ values ≥50%). *P* values less than 0.05 were considered statistically significant.

Publication bias was determined by using a method described by Egger et al [48]. The analysis of meta-regression and publication bias was performed using STATA version 10.0. For statistical heterogeneity, we explored reasons for using meta-regression, subgroup analysis and sensitivity analysis.

## References

[1] Rollig C, Illmer T (2009). The efficacy of arsenic trioxide for the treatment of relapsed and refractory multiple myeloma: a systematic review. Cancer Treat Rev.

[2] Shank BR, Brown VT, Schwartz RN (2015). Multiple myeloma maintenance therapy: a review of the pharmacologic treatment. J Oncol Pharm Pract.

[3] Latif T, Chauhan N, Khan R, Moran A, Usmani SZ (2012). Thalidomide and its analogues in the treatment of multiple myeloma. Exp Hematol Oncol.

[4] Yang B, Yu RL, Chi XH, Lu XC (2013). Lenalidomide treatment for multiple myeloma: systematic review and meta-analysis of randomized controlled trials. PLos One.

[5] Romano A, Conticello C, Di Raimondo F (2013). Bortezomib for the treatment of previously untreated multiple myeloma. Immunotherapy.

[6] Avet-Loiseau H, Attal M, Moreau P, Charbonnel C, Garban F, Hulin C, Leyvraz S, Michallet M, Yakoub-Agha I, Garderet L, Marit G, Michaux L, Voillat L (2007). Genetic abnormalities and survival in multiple myeloma: the experience of the Intergroupe Francophone du Myelome. Blood.

[7] Blade J, Rosinol L, Cibeira MT (2008). Prognostic factors for multiple myeloma in the era of novel agents. Ann Oncol.

[8] Smadja NV, Bastard C, Brigaudeau C, Leroux D, Fruchart C (2001). Hypodiploidy is a major prognostic factor in multiple myeloma. Blood.

[9] Landgren O, Morgan GJ (2014). Biological frontiers in multiple myeloma: From biomarker identification to clinical practice. Clin Cancer Res.

[10] Sharma A, Heuck CJ, Fazzari MJ, Mehta J, Singhal S, Greally JM, Verma A (2010). DNA methylation alterations in multiple myeloma as a model for epigenetic changes in cancer. Wiley Interdiscip Rev Syst Biol Med.

[11] Chim CS, Kwong YL, Liang R (2008). Gene hypermethylation in multiple myeloma: lessons from a cancer pathway approach. Clin Lymphoma Myeloma.

[12] Nobori T, Miura K, Wu DJ, Lois A, Takabayashi K, Carson DA (1994). Deletions of the cyclin-dependent kinase-4 inhibitor gene in multiple human cancers. Nature.

[13] Okamoto A, Demetrick DJ, Spillare EA, Hagiwara K, Hussain SP, Bennett WP, Forrester K, Gerwin B, Serrano M, Beach DH, Harris CC (1994). Mutations and altered expression of p16INK4 in human cancer. Proc Natl Acad Sci U S A.

[14] Sherr CJ (1996). Cancer cell cycles. Science.

[15] San-Miguel J, Garcia-Sanz R, Lopez-Perez R (2005). Analysis of methylation pattern in multiple myeloma. Acta Haematol.

[16] Kim H, Jekarl DW, Kim M, Kim Y, Lim J, Han K, Min CK (2013). Prevalence of p16 methylation and prognostic factors in plasma cell myeloma at a single institution in Korea. Ann Lab Med.

[17] Park G, Kang SH, Lee JH, Suh C, Kim M, Park SM, Kim TY, Oh B, Min HJ, Yoon SS, Yang IC, Cho HI, Lee DS (2011). Concurrent p16 methylation pattern as an adverse prognostic factor in multiple myeloma: a methylation-specific polymerase chain reaction study using two different primer sets. Ann Hematol.

[18] Gonzalez-Paz N, Chng WJ, McClure RF, Blood E, Oken MM, Van NB, James CD, Kurtin PJ, Henderson K, Ahmann GJ, Gertz M, Lacy M (2007). Tumor suppressor p16 methylation in multiple myeloma: biological and clinical implications. Blood.

[19] Fu W, Gao W, Hou J, Wang D, Ding S, Chen Q (2002). Hypermethylation of CPG island of p15 and p16 genes in multiple myeloma. Cancer Prev Cure Res.

[20] Delpu Y, Cordelier P, Cho WC, Torrisani J (2013). DNA methylation and cancer diagnosis. Int J Mol Sci.

[21] Ma X, Wang YW, Zhang MQ, Gazdar AF (2013). DNA methylation data analysis and its application to cancer research. Epigenomics.

[22] Fukushige S, Horii A (2013). DNA methylation in cancer: a gene silencing mechanism and the clinical potential of its biomarkers. Tohoku J Exp Med.

[23] Galm O, Wilop S, Reichelt J, Jost E, Gehbauer G, Herman JG, Osieka R (2004). DNA methylation changes in multiple myeloma. Leukemia.

[24] Guillerm G, Depil S, Wolowiec D, Quesnel B (2003). Different prognostic values of p15(INK4b) and p16(INK4a) gene methylations in multiple myeloma. Haematologica.

[25] Seidl S, Ackermann J, Kaufmann H, Keck A, Nosslinger T, Zielinski CC, Drach J, Zochbauer-Muller S (2004). DNA-methylation analysis identifies the E-cadherin gene as a potential marker of disease progression in patients with monoclonal gammopathies. Cancer.

[26] Uchida T, Kinoshita T, Ohno T, Ohashi H, Nagai H, Saito H (2001). Hypermethylation of p16INK4A gene promoter during the progression of plasma cell dyscrasia. Leukemia.

[27] Long C, Yin B, Lu Q, Zhou X, Hu J, Yang Y, Yu F, Yuan Y (2007). Promoter hypermethylation of the RUNX3 gene in esophageal squamous cell carcinoma. Cancer Invest.

[28] Tonomoto Y, Tachibana M, Dhar DK, Onoda T, Hata K, Ohnuma H, Tanaka T, Nagasue N (2007). Differential expression of RUNX genes in human esophageal squamous cell carcinoma: downregulation of RUNX3 worsens patient prognosis. Oncology.

[29] Watanabe T, Kobunai T, Ikeuchi H, Yamamoto Y, Matsuda K, Ishihara S, Nozawa K, Iinuma H, Kanazawa T, Tanaka T, Yokoyama T, Konishi T, Eshima K (2011). RUNX3 copy number predicts the development of UC-associated colorectal cancer. Int J Oncol.

[30] Goel A, Arnold CN, Tassone P, Chang DK, Niedzwiecki D, Dowell JM, Wasserman L, Compton C, Mayer RJ, Bertagnolli MM, Boland CR (2004). Epigenetic inactivation of RUNX3 in microsatellite unstable sporadic colon cancers. Int J Cancer.

[31] Ito K, Lim AC, Salto-Tellez M, Motoda L, Osato M, Chuang LS, Lee CW, Voon DC, Koo JK, Wang H, Fukamachi H, Ito Y (2008). RUNX3 attenuates beta-catenin/T cell factors in intestinal tumorigenesis. Cancer Cell.

[32] Chen W, Gao N, Shen Y, Cen JN (2010). Hypermethylation downregulates Runx3 gene expression and its restoration suppresses gastric epithelial cell growth by inducing p27 and caspase3 in human gastric cancer. J Gastroenterol Hepatol.

[33] Dib A, Barlogie B, Shaughnessy JD, Kuehl WM (2007). Methylation and expression of the p16INK4A tumor suppressor gene in multiple myeloma. Blood.

[34] Urashima M, Teoh G, Ogata A, Chauhan D, Treon SP, Sugimoto Y, Kaihara C, Matsuzaki M, Hoshi Y, DeCaprio JA, Anderson KC (1997). Characterization of p16(INK4A) expression in multiple myeloma and plasma cell leukemia. Clin Cancer Res.

[35] Du HL, Qi Y, Shi YJ, Bu DF, Wu SL (2002). [Apoptosis and re-expression of p16 gene in the myeloma cell line U266 induced by synergy of histone deacetylase inhibitor and demethylating agent]. [Article in Chinese]. Ai Zheng.

[36] Fu HY, Shen JZ (2005). [Hypermethylation of CpG island of p16 gene and arsenic trioxide induced p16 gene demethylation in multiple myeloma]. [Article in Chinese]. Zhonghua Nei Ke Za Zhi.

[37] Landgren O (2013). Monoclonal gammopathy of undetermined significance and smoldering multiple myeloma: biological insights and early treatment strategies. Hematology Am Soc Hematol Educ Program.

[38] Agarwal A, Ghobrial IM (2013). Monoclonal gammopathy of undetermined significance and smoldering multiple myeloma: a review of the current understanding of epidemiology, biology, risk stratification, and management of myeloma precursor disease. Clin Cancer Res.

[39] Greenberg AJ, Rajkumar SV, Vachon CM (2012). Familial monoclonal gammopathy of undetermined significance and multiple myeloma: epidemiology, risk factors, and biological characteristics. Blood.

[40] Iida S, Ueda R (2003). Multistep tumorigenesis of multiple myeloma: its molecular delineation. Int J Hematol.

[41] Stanganelli C, Arbelbide J, Fantl DB, Corrado C, Slavutsky I (2010). DNA methylation analysis of tumor suppressor genes in monoclonal gammopathy of undetermined significance. Ann Hematol.

[42] Chim CS, Liang R, Leung MH, Kwong YL (2007). Aberrant gene methylation implicated in the progression of monoclonal gammopathy of undetermined significance to multiple myeloma. J Clin Pathol.

[43] McShane LM, Altman DG, Sauerbrei W, Taube SE, Gion M, Clark GM (2005). Reporting recommendations for tumor marker prognostic studies (REMARK). J Natl Cancer Inst.

[44] Steels E, Paesmans M, Berghmans T, Branle F, Lemaitre F, Mascaux C, Meert AP, Vallot F, Lafitte JJ, Sculier JP (2001). Role of p53 as a prognostic factor for survival in lung cancer: a systematic review of the literature with a meta-analysis. Eur Respir J.

[45] DerSimonian R, Laird N (1986). Meta-analysis in clinical trials. Control Clin Trials.

[46] Higgins JP, Thompson SG, Deeks JJ, Altman DG (2003). Measuring inconsistency in meta-analyses. BMJ.

[47] DerSimonian R (1996). Meta-analysis in the design and monitoring of clinical trials. Stat Med.

[48] Egger M, Davey Smith G, Schneider M, Minder C (1997). Bias in meta-analysis detected by a simple, graphical test. BMJ.

[49] Braggio E, Maiolino A, Gouveia ME, Magalhaes R, Souto Filho JT, Garnica M, Nucci M, Renault IZ (2010). Methylation status of nine tumor suppressor genes in multiple myeloma. Int J Hematol.

[50] Hatzimichael E, Benetatos L, Dasoula A, Dranitsaris G, Tsiara S, Georgiou I, Syrrou M, Stebbing J, Coley HM, Crook T, Bourantas KL (2009). Absence of methylation-dependent transcriptional silencing in TP73 irrespective of the methylation status of the CDKN2A CpG island in plasma cell neoplasia. Leuk Res.

[51] Martin P, Garcia-Cosio M, Santon A, Bellas C (2008). Aberrant gene promoter methylation in plasma cell dyscrasias. Exp Mol Pathol.

[52] Liang X, Jiang Y, Xu W, Liu J (2006). Study on hypermethylation and mRNA expression of p16 and p15 gene in multiple myeloma Clinical Focus.

[53] Chim CS, Kwong YL, Fung TK, Liang R (2004). Methylation profiling in multiple myeloma. Leuk Res.

[54] Kramer A, Schultheis B, Bergmann J, Willer A, Hegenbart U, Ho AD, Goldschmidt H, Hehlmann R (2002). Alterations of the cyclin D1/pRb/p16(INK4A) pathway in multiple myeloma. Leukemia.

[55] Chim CS, Fung TK, Liang R (2003). Disruption of INK4/CDK/Rb cell cycle pathway by gene hypermethylation in multiple myeloma and MGUS. Leukemia.

[56] Guillerm G, Gyan E, Wolowiec D, Facon T, Avet-Loiseau H, Kuliczkowski K, Bauters F, Fenaux P, Quesnel B (2001). p16(INK4a) and p15(INK4b) gene methylations in plasma cells from monoclonal gammopathy of undetermined significance. Blood.

[57] Ng MH, Chung YF, Lo KW, Wickham NW, Lee JC, Huang DP (1997). Frequent hypermethylation of p16 and p15 genes in multiple myeloma. Blood.

[58] Tasaka T, Asou H, Munker R, Said JW, Berenson J, Vescio RA, Nagai M, Takahara J, Koeffler HP (1998). Methylation of the p16INK4A gene in multiple myeloma. Br J Haematol.

